# CCR7上调MMP-9表达促进非小细胞肺癌转移

**DOI:** 10.3779/j.issn.1009-3419.2010.11.04

**Published:** 2010-11-20

**Authors:** 洋 李, 巍 刘, 莉 方, 娟 南, 占雀 张, 清华 周

**Affiliations:** 1 300052 天津，天津医科大学总医院，天津市肺癌研究所，天津市肺癌转移与肿瘤微环境实验室 Tianjin Key Laboratory of Lung Cancer Metastasis and Tumor Microenviroment, Tianjin Lung Cancer Institute, Tianjin Medical Univercity General Hospital, Tianjin 300052, China; 2 300191 天津，天津药物研究院，天津市新药设计与发现重点实验室 Tianjin Key Laboratory of Molecular Design and Drug Discovery, Tianjin Institute of Pharmaceutical Research, Tianjin 300193, China; 3 110001 沈阳，中国医科大学附属第一医院肝胆外科 Department of Hepticbile Surgery, the First Hospital Affiliated to China Medical University, Shenyang 110001, China; 4 044600 芮城，山西省芮城县人民医院 Ruicheng People's Hospital, Shanxi 044600, China

**Keywords:** CCR7, MMP-9, 肺肿瘤, 转移, CCR7, MMP-9, Lung neoplasms, Metastasis

## Abstract

**背景与目的:**

趋化因子激素受体（CC chemokine receptor 7, CCR7）与非小细胞肺癌（non-small cell lung cancer, NSCLC）的淋巴结转移密切相关，但CCR7促进其淋巴结转移的机制尚不明了。本研究通过观察CCR7和MMP-9在NSCLC组织中的表达和相互关系，探讨CCR7促进NSCLC淋巴结转移的机制。

**方法:**

应用免疫组织化学染色（SP法）检测90例NSCLC组织中CCR7、MMP-9的表达；将BE1细胞经趋化因子CCL19处理24 h后，应用RTPCR和Western blot方法检测MMP-9 mRNA和蛋白表达水平。

**结果:**

免疫组织化学结果显示：CCR7主要表达于癌细胞胞质和（或）胞膜，MMP-9主要表达于癌细胞胞质，NSCLC中CCR7、MMP-9阳性表达率分别为70%（63/90）和65.5%（59/90），*χ*^2^检验显示CCR7和MMP-9表达与NSCLC的临床病理分期（*P*=0.003, *P*=0.001）和淋巴结转移（*P*=0.004, *P*=0.003）密切相关，而与年龄、组织学类型、分化程度无关（*P* > 0.05）。此外，CCR7和MMP-9表达正相关（*r*=0.342, *P*=0.001）。CCL21处理组BE1细胞后MMP-9 mRNA和蛋白水平均上调（*P* < 0.05）。

**结论:**

CCR7和MMP-9表达与NSCLC侵袭转移密切相关，CCL19/CCR7通过上调NSCLC中MMP-9表达促进其转移。

## Abstract (en)

**Background and objective:**

It has been proven that CC chemokine receptor 7 (CCR7) is closely related with the lymph node metastasis of non-small cell lung cancer (NSCLC), but the mechanism of NSCLC metastasis is not very clear. The aim of this study is to investigate the expressions of CCR7 and MMP-9 in NSCLC and the relationship of their expressions, and to explore the mechanism of CCR7 promoting NSCLC metastasis.

**Methods:**

The expressions of CCR7 and MMP-9 protein were detected in 90 specimens of human primary NSCLC by immunohistochemical SP method. Human large lung cell line BE1 cells were pre-incubated with CCL19 for 24 h; the changes of MMP-9 were detected by RT-PCR and Western blot.

**Results:**

Immunohistochemistry showed that CCR7 was distributed in cytoplasm and/or membrane of tumor cells and MMP-9 was distributed in cytoplasm of cancer cells. The expressions of CCR7 and MMP-9 protein were found to be 70% (63/90) and 65.5% (59/90) in NSCLC, respectively. The expressions of CCR7 and MMP-9 protein were closely related to the clinical stages (*P*=0.003, *P*=0.001) and lymph node metastasis (*P*=0.004, *P*=0.003) of NSCLC, but there was no correlation with age, gender, histology (*P* > 0.05). Furthermore, a significant correlation was found between CCR7 and MMP-9 (*r*=0.342, *P*=0.001). In addition, the expressions of MMP-9 mRNA and protein levels were increased in CCL19 pre-incubated group (*P* < 0.05).

**Conclusion:**

The expressions of CCR7 and MMP-9 are significantly associated with NSCLC invasion and metastasis. The upregulation of MMP-9 is regulated by CCR7 in NSCLC.

趋化因子激素受体（CC chemokine receptor 7, CCR7）是CC家族趋化因子受体的成员之一，近来研究^[1-3]^发现，CCR7在多种肿瘤细胞中过表达，CCR7与其配体CCL21相互作用在肿瘤的特异性器官转移中发挥重要作用，然而CCR7促进非小细胞肺癌（non-small cell lung cancer, NSCLC）转移的机制却不十分清楚。肿瘤的侵袭和转移是一个十分复杂的过程，肿瘤细胞的侵袭一般要经历细胞骨架改变、粘附性丧失、运动性的增强和表达溶解性蛋白酶等过程，此过程与基质金属蛋白酶9（matrix metalloproteinase 9, MMP-9）有着密不可分的关系^[4]^。近来，有研究^[5, 6]^发现CCR7可以上调结肠癌SW480细胞及慢性B细胞淋巴瘤细胞中MMP-9的表达。本实验拟探讨CCR7、MMP-9在NSCLC中的表达情况及相互关系。

## 1 材料与方法

### 1.1 标本来源

90例NSCLC组织腊块来自中国医科大学附属第一医院1980年1月-2005年12月间手术切除的肺癌组织，病史回顾无其它恶性肿瘤史。所有标本均经病理确诊。所有标本均经10%福尔马林液固定，石蜡包埋，行4 μm连续切片，备用。

### 1.2 临床病理资料

90例NSCLC包括：年龄≤50岁者50例，≥50岁者40例；鳞癌55例，腺癌35例；临床病理分期显示Ⅰ期、Ⅱ期共39例，Ⅲ期、Ⅳ期共51例；淋巴结转移54例，无淋巴结转移36例。所有患者术前均未接受过任何的放疗和化疗，术后均经两个疗程的系统化疗。

### 1.3 免疫组织化学染色

羊抗人CCR7多克隆抗体购自Santa Cruz公司，鼠抗人MMP-9单克隆抗体购自R&D公司；SP免疫组化试剂盒、DAB酶底物显色试剂盒均购自福州迈新生物技术开发公司。染色方法按试剂盒说明书步骤进行，用PBS代替一抗作为阴性对照。

### 1.4 免疫组化结果判断标准

结果判定在双盲法下进行，每张切片由两名病理医师分别判断。CCR7以胞浆和/或胞膜出现棕黄色颗粒视为阳性细胞；MMP-9以胞浆出现棕黄色颗粒视为阳性细胞。至少5个高倍视野下（× 400）评价CCR7和MMP-9染色强度（1分=弱分，2分=强）和阳性肿瘤细胞百分比（0分=阴性，1%-50%=1分，51%-75%=2分，≥76%=3分）。上述两项评分的乘积作为每个标本染色的最终评分，最后确定肺癌标本的染色情况分别为低表达（评分≤3分）或高表达（评分>3分）。

### 1.5 细胞培养

本研究采用人大细胞肺癌细胞系BE1，细胞用含10%新鲜小牛血清的DMEM（Gibco, USA）、100 U/mL的青霉素、100 U/mL的链霉素的培养基，在37 ℃、5%CO_2_的条件下培养。

### 1.6 RT-PCR检测MMP-9 mRNA表达

取对数生长期的细胞用Trizol（Invitrogen, USA）提取总RNA。逆转录条件为30 ℃、10 min，42 ℃、40 min，99 ℃、5 min，5 ℃、5 min。PCR反应条件为：94 ℃、5 min，94 ℃、40 s，55 ℃、40 s，72 ℃、40 s，72 ℃、5 min。取扩增产物5 μL，1.5%琼脂糖凝胶电泳，用Image J软件进行表达强度分析。引物均由辽宁博春天生物技术有限公司合成。PCR引物为：MMP-9上游5’-GCC ACT TGT CGG CGA TAA GG-3’；下游5’-CAC TGT CCA CCC CTC AGA GC-3’，243 bp。β-actin上游5’-AAA TCG TGC GTG ACA TTA A-3’；下游5’-CTC GTC ATA CTC CTG CTT G-3’，513 bp。

### 1.7 Western blot检测MMP-9蛋白表达

收集细胞提取总蛋白。采用考马斯亮蓝法进行蛋白定量。取等量蛋白，进行SDS-聚丙烯酰胺凝胶电泳和转印，一抗（anti-CCR7 1:200稀释，anti-MMP-9 1:100稀释）4 ℃孵育过夜，各自对应二抗（1:1 500稀释）37 ℃孵育2 h，最后DAB显色。EC3 Imaging System凝胶成像系统采集图像，Image J软件用于半定量分析特异性条带的光密度值。实验至少重复3次，取平均值。

### 1.8 统计学分析

采用SPSS 13.0统计学分析软件，数据采用Mean±SD表示，对CCR7和MMP-9表达与临床病理因素的关系用*χ*^2^检验；CCR7与MMP-9表达的关系采用*Spearman*等级相关分析，*P* < 0.05为有统计学差异。

## 2 结果

### 2.1 CCR7和MMP-9蛋白的免疫组化检测结果CCR7主要

表达于肿瘤细胞的胞质和（或）胞膜（图 1B），CCR7阳性率为70%（63/90）。MMP-9主要表达于癌细胞胞质（图 1D），MMP-9阳性表达率为65.5%（59/90）。CCR7和MMP-9在有淋巴结转移的肿瘤组织中高表达（图 1B、图 1D），而在无淋巴结转移的肿瘤组织中低表达（图 1A、图 1C）。对CCR7和MMP-9的表达情况及其与NSCLC临床病理特征的相关性进行统计分析，结果显示：CCR7和MMP-9与NSCLC的临床病理分期（*P*=0.003, *P*=0.001）和淋巴结转移（*P*=0.004, *P*=0.003）有密切关系，而与年龄、组织学类型、分化程度无关（*P*>0.05）（表 1）。

**1:**
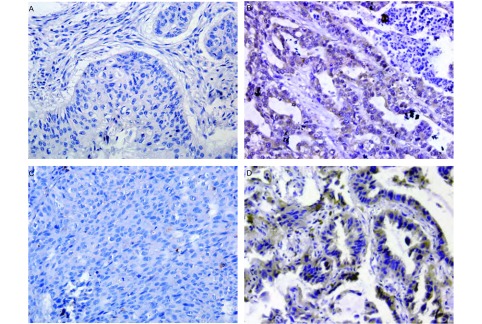
90例非小细胞肺癌患者中CCR7和MMP-9的表达（SP，×200）。A和C为无淋巴结转移组；B和D为有淋巴结转移组。CCR7阴性表达（A），CCR7阳性表达（B），MMP-9阴性表达（C），MMP-9阳性表达（D）。 The expressions of CCR7 and MMP-9 protein were detected in 90 specimens of human primary non-small cell lung cancer (SP, ×200). A and C are negative for lymphatic metastasis; B and D are positive for lymphatic metastasis. CCR7 negative expression (A), CCR7 positive expression (B), MMP-9 negative expression (C), MMP-9 positive expression (D).

**1:** CCR7和MMP-9在非小细胞肺癌中的表达及其与临床病理参数的关系 Correlation of clinicopathologic parameters with CCR7 and MMP-9 expressions in non-small cell lung cancer

Factors	*n*	Expression of CCR7		*P*	Expression of MMP-9		*P*
	Lower	Higher	Lower		Higher		
Age				0.355			0.215
< 50	50	13	37		20	30	
≤50	40	14	26		11	29	
Histology				0.813			0.980
Squamous cell carcinoma	55	17	38		19	36	
Adenocarcinoma	35	10	25		12	23	
Differentiation				0.899			0.913
Well	14	4	10		5	9	
Poor-moderate	76	23	53		26	50	
Stage				0.003			0.001
Ⅰ+Ⅱ	39	18	21		21	18	
Ⅲ+Ⅳ	51	9	42		10	41	
Lymph node status				0.004			0.003
Negative	36	17	19		19	17	
Positive	54	10	44		12	42	

### 2.2 NSCLC中CCR7的表达与MMP-9的关系

对CCR7和MMP-9低表达和高表达的数据进行比较，结果发现：CCR7与MMP-9的表达相关（*r*=0.342, *P* < 0.05）（表 2）。

**2:** 非小细胞肺癌中MMP-9表达与CCR7表达的关系 Relationship of the expressions of MMP-9 with CCR7

MMP-9	CCR7		*r*	*P*
	Lower	Higher		
Lower	16	15	0.342	0.001
Higher	11	48		

### 2.3 CCL21/CCR7上调BE1细胞中MMP-9的表达

为了阐明MMP-9是否为CCR7的下游效应分子，我们将BE1细胞分别经20 ng/mL和100 ng/mL CCL21处理24 h后，通过RTPCR和Western blot方法检测MMP-9表达。结果表明，与对照组相比，CCL21处理组MMP-9 mRNA和蛋白水平均上调（*P* < 0.05），且具有剂量依赖性（图 2）。这些结果表明CCL21/CCR7可以上调肺癌BE1细胞中MMP-9的表达。

**2:**
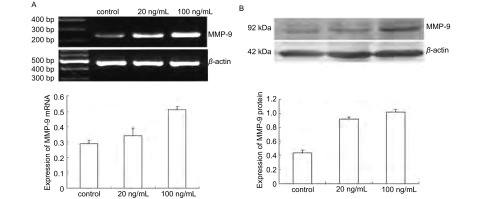
CCR7调控BE1细胞中MMP-9的表达。经CCL21处理后BE1细胞中MMP-9 mRNA（A）和蛋白（B）水平的表达情况。 The expression of MMP-9 was regulated by CCR7 in BE1 cells. Expression of MMP-9 mRNA (A) and protein (B) in BE1 cells after CCL21 stimulation.

## 3 讨论

CCR7是CC家族趋化因子受体成员之一，通过与其配体CCL21相互作用在淋巴细胞的归巢过程中起重要作用。近来研究发现，CCR7在NSCLC^[1, 7, 8]^、乳腺癌^[2]^、头颈部鳞癌^[3]^等多种肿瘤细胞中过表达，并且与这些肿瘤的淋巴结转移密切相关。

肿瘤的侵袭和转移是一个多因素、多步骤的复杂过程。MMP介导的细胞外基质及基底膜的降解是肿瘤侵袭转移的一个关键步骤。研究^[9-11]^表明MMP-9在多种恶性肿瘤组织、培养的肿瘤细胞及癌基因转化细胞中表达增强，体外侵袭实验证实肿瘤细胞的高侵袭能力与MMP-9的表达增强有关。近来有研究^[5, 6]^报道CCL21/CCR7可以调控结肠癌SW480细胞及慢性B细胞淋巴瘤细胞中MMP-9表达。基于以上理论基础，我们认为NSCLC中CCR7可能上调MMP-9表达从而影响肿瘤的转移。为此，我们运用免疫组化方法检测了90例NSCLC组织中CCR7和MMP-9的表达。结果显示CCR7与NSCLC患者的临床分期和淋巴结转移密切相关，这与Takanami^[1]^的报道相一致；此外，我们还发现CCR7高表达组MMP-9高表达，统计学分析表明CCR7与MMP-9蛋白表达呈正相关（*P* < 0.05）。上述结果表明CCR7可能通过上调肿瘤细胞中MMP-9的表达影响肿瘤转移。

为了进一步阐明CCR7是否可上调NSCLC中MMP-9的表达，我们将BE1细胞经CCL21刺激24 h后发现，MMP-9的mRNA和蛋白表达水平明显上调。因此，我们认为CCR7是诱导MMP-9表达的重要调节因子，但CCR7在NSCLC进展中调控MMP-9表达的精确机制有待更进一步的研究。

综上所述，CCR7可以调控NSCLC中MMP-9的表达从而影响肿瘤的淋巴结转移，同时本实验也为MMP-9和CCR7成为NSCLC干预治疗靶点提供了新的有力证据。

## References

[1] Takanami I (2003). Overexpression of CCR7 mRNA in non small cell lung cancer: correlation with lymph node metastasis. Int J Cancer.

[2] Müller A, Homey B, Soto H (2001). Involvement of chemokine receptors in breast cancer metastasis. Nature.

[3] Wang J, Xi L, Hunt JL, Gooding W (2004). Expression pattern of chemokine receptor 6 (CCR6) and CCR7 in squamous cell carcinoma of the head and neck identifies a novel metastatic phenotype. Cancer Res.

[4] Zheng H, Takahashi H, Murai Y (2006). Expressions of MMP-2, MMP-9 and VEGF are closely linked to growth, invasion, metastasis and angiogenesis of gastric carcinoma. Anticancer Res.

[b5] 5Li J, Sun R, Tao K, *et al*. The CCL21/CCR7 pathway plays a key role in human colon cancer metastasis through regulation of matrix metalloproteinase-9. Dig Liver Dis. 2010 Jul 5. [Epub ahead of print]

[6] Redondo-Muñoz J, José Terol M, García-Marco JA (2008). Matrix metalloproteinase-9 is up-regulated by CCL21/CCR7 interaction via extracellular signal-regulated kinase-1/2 signaling and is involved in CCL21- driven B-cell chronic lymphocytic leukemia cell invasion and migration. Blood.

[7] Maekawa S, Iwasaki A, Shirakusa T (2008). Association between the expression of chemokine receptors CCR7 and CXCR3, and lymph node metastatic potential in lung adenocarcinoma. Oncol Rep.

[8] Lau SK, Boutros PC, Pintilie M (2007). Three-gene prognostic classifier for early-stage non small-cell lung cancer. J Clin Oncol.

[9] Sato H, Seiki M (1993). Regulatory mechanism of 92 kDa type Ⅳ collagenase gene expression which is associated with invasiveness of tumor cells. Oncogene.

[10] Strup-Perrot C, Vozenin-Brotons MC, Vandamme M (2006). Expression and activation of MMP -2, -3, -9, -14 are induced in rat colon after abdominal X-irradiation. Scand J Gastroenterol.

[11] Ogata Y, Matono K, Nakajima M (2006). Efficacy of the MMP inhibitor MMI270 against lung metastasis following removal of orthotopically transplanted human colon cancer in rat. Int J Cancer.

